# Traditional Chinese Medicine and Natural Products: Potential Approaches for Inflammatory Bowel Disease

**DOI:** 10.3389/fphar.2022.892790

**Published:** 2022-07-07

**Authors:** Shuo Yuan, You Li, Jiao Li, Jia-Chen Xue, Qi Wang, Xiao-Ting Hou, Huan Meng, Ji-Xing Nan, Qing-Gao Zhang

**Affiliations:** ^1^ Key Laboratory of Natural Medicines of the Changbai Mountain, Ministry of Education, College of Pharmacy, Yanbian University, Yanji, China; ^2^ Chronic Disease Research Center, Medical College, Dalian University, Dalian, China; ^3^ Department of Immunology and Pathogenic Biology, Yanbian University College of Basic Medicine, Yanji, China

**Keywords:** inflammatory bowel disease, traditional Chinese medicine, natural product, immunity, gut microbes

## Abstract

Inflammatory bowel disease (IBD) is a rare, recurrent, and intractable inflammation obstruction of the stomach tract, usually accompanied by inflammation of cell proliferation and inflammation of the colon and carries a particular cause of inflammation. The clinical use of drugs in western countries affects IBD treatment, but various adverse effects and high prices limit their application. For these reasons, Traditional Chinese Medicine (TCM) is more advantageous in treating IBD. This paper reviews the mechanism and research status of TCM and natural products in IBD treatment by analyzing the relevant literature to provide a scientific and theoretical basis for IBD treatment.

## 1 Introduction

Inflammatory bowel disease (IBD) is a group of chronic recurrent inflammatory diseases, mainly Crohn’s disease (CD) and ulcerative colitis (UC). Among them, UC especially involves the colon, while CD can affect the entire gastric intestinal tract from the oral to the anal canal and has a jumping distribution (Kaplan, 2015). The recurrent and disabling nature of IBD can seriously affect people’s quality of life. The global prevalence of IBD has also increased year by year (Ng et al., 2013). The pathogenesis of IBD is not fully understood. Current studies suggest that it may be related to infection, environment, genetics, immunity, and intestinal microorganisms, among which abnormal immune function has received increasing attention in the pathogenesis of IBD (Zhao et al., 2013b).

Chinese medicine is an ancient medicine with a complete medical system, which mainly treats diseases through evidence-based treatment. Traditional Chinese Medicine (TCM) has contributed to Chinese medicine, characterized by comprehensive resources and low cost (Bu et al., 2020). China is a vast country with abundant TCM resources, easy to grow, harvest, and use. TCM is often boiled into a tonic to treat diseases. It is boiled according to different medicinal properties and parts of the drug, so its active ingredients are not easily lost, and its side effects are relatively small. The main component of TCM is herbal, and it has its unique function and treatment. Therefore, it can be used flexibly by adding or subtracting according to the symptoms and the person thus (Wang WY. et al., 2021). In addition, a large number of natural products found in TCM are gaining attention due to their unique advantages of low adverse effects, stable efficacy, and wide range of access and targets.

With the increased interest in TCM worldwide, many advances have been made in treating IBD with TCM and natural products. The relevant research progress of TCM and natural products in the treatment of IBD is summarized to provide ideas and methods for TCM to prevent and treat IBD.

## 2 The Pathogenesis of IBD

### 2.1 Immune Cells

#### 2.1.1 Macrophages

**FIGURE 1:**
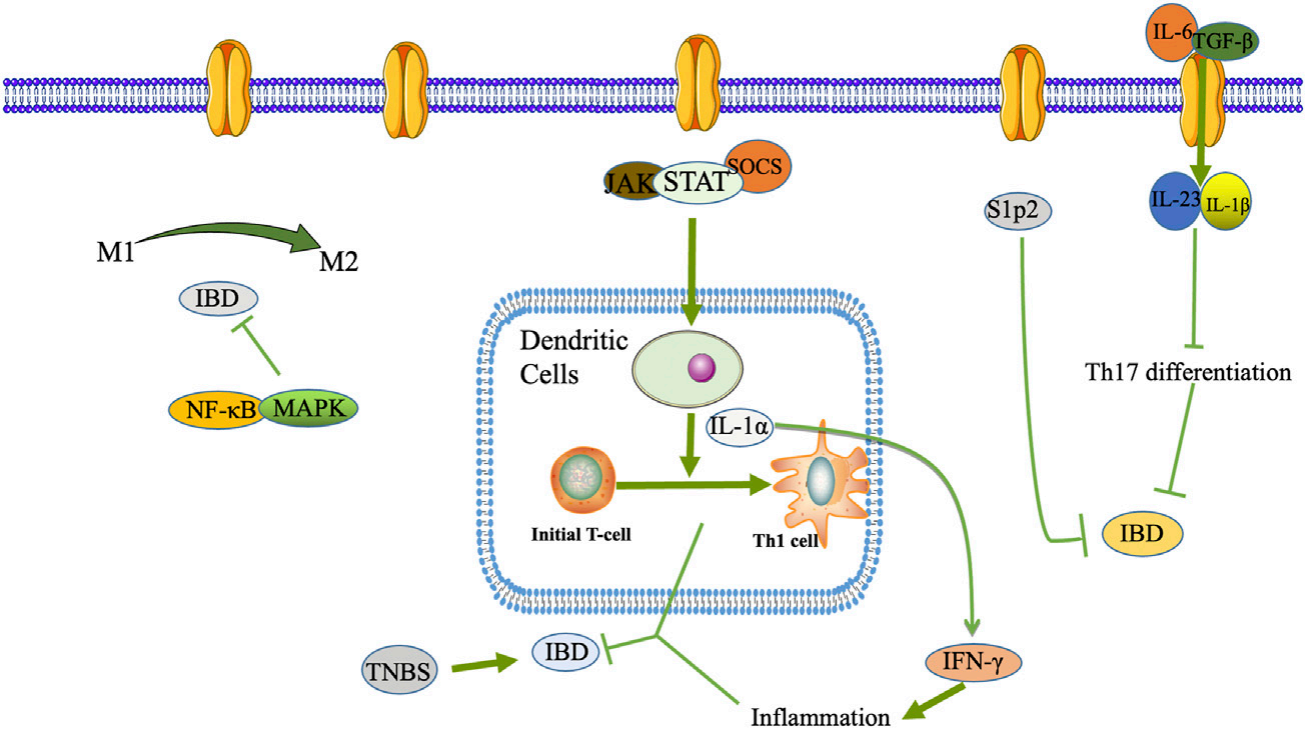
DCs, macrophages, T lymphocyte differentiation and Th17/Treg transformation play a role in the pathogenesis of IBD.

As shown in Figure 1, macrophages are monocytes that may play a phagocytic function in immune regulation, and they play an essential role in the innate and specific immune response (Suzuki et al., 2016). Macrophages will initiate various cell polarization pathways upon stimulation with different cytokines, chemokines, and signaling molecules (Saradna et al., 2018). Macrophages were first discovered to trigger all immune responses, including T-cell, B-cell adaptive immune responses, polarization-related Th1, and Th2 cytokines. Therefore, macrophages were divided into M1 and M2 macrophages (Mills, 2015). M1 macrophages, also known as classically activated macrophages, have a pro-inflammatory effect, while M2 macrophages, also known as alternatively activated macrophages, exert an anti-inflammatory influence and can be subdivided into different subtypes depending on the activating factor: M2a, M2b, M2c, and M2d (Atri et al., 2018). M1 macrophages are predominantly expressed at the onset of inflammation and secrete many pro-inflammatory factors, which promote the intensification of the inflammatory response.

In contrast, to counteract the excessive inflammatory response at a later stage, macrophages can be converted from M1 to M2 phenotype to suppress inflammation protecting the host from extreme damage and promoting wound healing. Since the pro-inflammatory effect of M1 macrophages and the anti-inflammatory effect of M2 macrophages have opposite roles in the pathogenesis of IBD, regulation of macrophage polarization may be effective in alleviating IBD (Lin et al., 2014). Wang SW. et al. (2019) used DSS-induced colitis mice to reveal the intrinsic mechanism of Cinobufacini ameliorates colitis. It was found that Cinobufacini significantly reduced the number of M1 macrophages and increased the number of M2 macrophages, and inhibited the polarization of M1 macrophages in lipopolysaccharide-induced RAW 264.7 cells.

#### 2.1.2 T Lymphocytes

**FIGURE 2:**
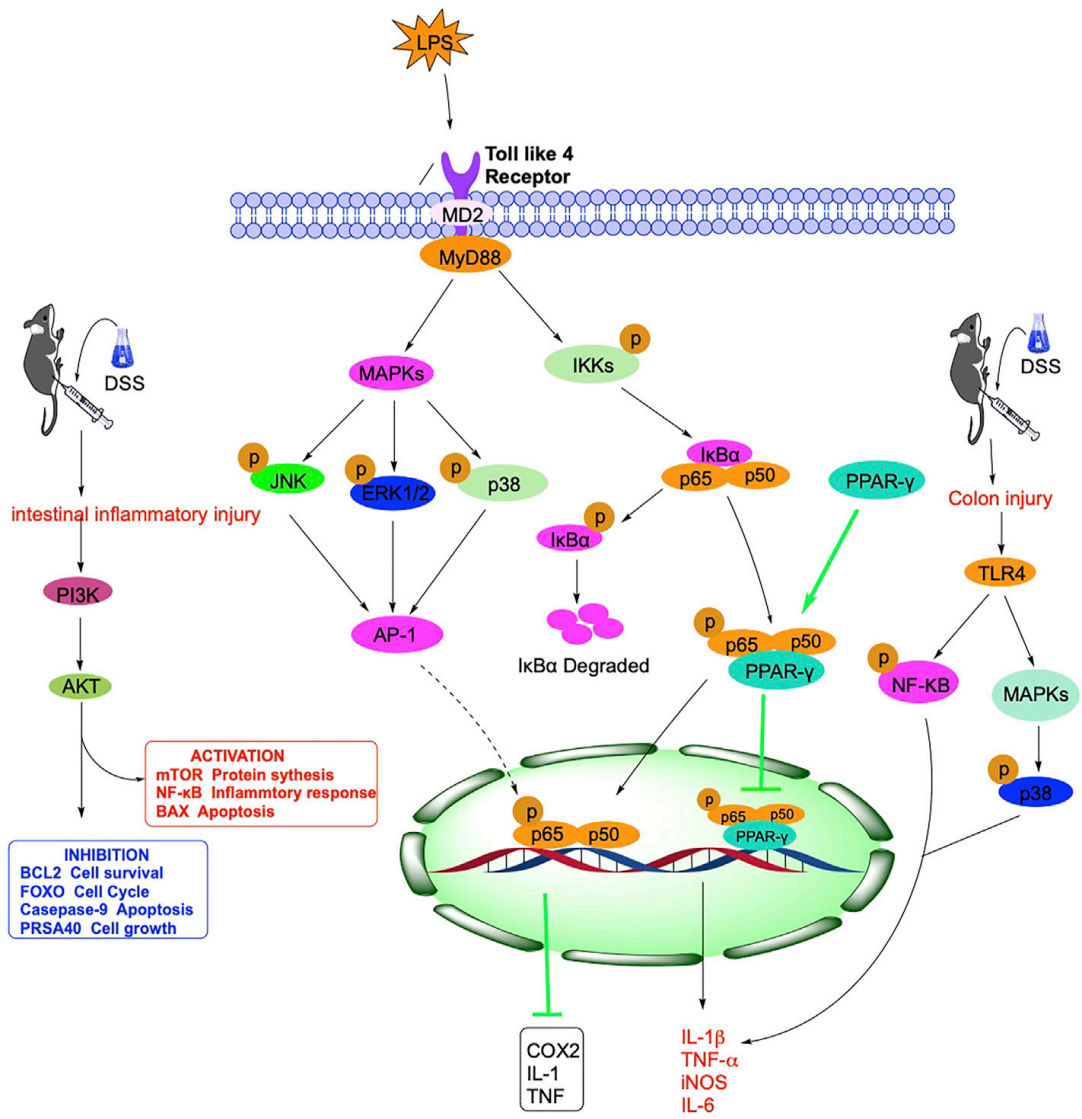
Mechanism of NF-кB, TLR4, PPAR, PI3K and other signaling pathways related in IBD.

During the development and maturation of the thymus, T lymphocytes differentiate into CD4^+^ T lymphocytes and CD8^+^ T lymphocytes. CD4^+^ T lymphocytes are helper T cells (Th), which play a significant role in cellular immunity and assist humoral immunity. The intestinal inflammatory infiltrate consists mainly of CD4^+^ T cells, regulatory T cells (Tregs), and central memory T lymphocytes (Zhong et al., 2020). CD4^+^ T lymphocytes are enriched in the damaged tissues of IBD patients, and blocking or clearing CD4^+^ T lymphocytes is effective in IBD patients (Figure 2).

Typical T helper cell subsets are Th1, Th2, Th9, Th17, Th22, T follicular helper (Tfh), and auto Tregs (Zhong et al., 2020). Th17 cells play an essential barrier role in the skin and intestinal mucosa against bacteria and fungi and are imperative drivers of autoimmune disease, often exacerbated when triggered in the autoimmune environment. Th17 differentiation is mainly driven by interleukin (IL)-6 and transforming growth factor (TGF)-β, further stabilized by IL-23, IL-1β, and other co-factor signals (Wu and Wan, 2020). The role of Tregs cells is mainly to suppress inflammation by suppressing T cells and regulating other immune cells in their environment, especially interconnected with Th17 cells in terms of differentiation, which together maintain the balance of the body’s immune microenvironment. Once this balance is upset, various autoimmune diseases, including IBD, can occur (Haase et al., 2018). It is now generally accepted that Tregs are abundant in the intestine and are vital anti-inflammatory CD4^+^ T cells (Tanoue et al., 2016).

#### 2.1.3 Dendritic Cells

Intestinal dendritic cells (DCs), macrophages, intrinsic lymphoid-like cells, and neutrophils constitute the first line of defense of the inherent immune system of the intestine. They play an essential role in maintaining intestinal immune tolerance and the physiological state to generate intestinal inflammatory responses in pathological conditions. DCs serve as the most critical specialized antigen-presenting cells *in vivo*. In a mouse IBD model, DCs differentiate initial T cells into Th1 cells by secreting IL-12 and producing large amounts of interferon (IFN)-γ to mediate the intestinal mucosal inflammatory response (Kunkl et al., 2020) (Figure 1).

### 2.2 Signaling Pathways

Many botanical drugs and natural products have practical anti-inflammatory and immunomodulatory effects through various signaling pathways. They can inhibit the expression of various cytokines, such as tumor necrosis factor (TNF)-α, IL-1β, IL-4, IL-6, IL-8, IL-12, IL-18, IL-22, and inducible nitric oxide synthase (iNOS) (Zhao HM. et al., 2017; Li Y. et al., 2017; Zhao L. et al., 2017; Wang Y. et al., 2017; Huang et al., 2017; Sun et al., 2018; Wu et al., 2018; Lian et al., 2019; Ni et al., 2019; Yin et al., 2019; Liu et al., 2020; Wang Y. Z. et al., 2021; Yu et al., 2021).

#### 2.2.1 NF-кB

The body’s immune function dysfunction is vital in the pathogenesis of IBD, and Nuclear Factor Kappa B (NF-κB) can regulate some inflammatory factors. NF-κB was discovered in 1986 in nuclear extracts of B lymphocytes, where p50 and p65 subunits form a heterodimer that is a crucial component of the classical NF-κB signaling pathway. In normal cells, p50/p65 is mainly bound to the NF-κB inhibitor protein IκB (inhibitor of NF-κB) in an inactivated form (Ward et al., 2015). It has been demonstrated that NF-κB is a pleiotropic signaling transcription factor, which can participate in inflammatory responses, immune responses, apoptosis, and other changes in the body when activated by various factors such as chemokines and oxidative stress (McDaniel et al., 2016). As an essential ligand-activated transcription factor, mouse pregnane X receptor (PXR) is expressed in the liver and intestine of mammals, and its activation protects mice from IBD. Alantolactone and Alpinetin, isolated from *Inula helenium* L., *Inula japonica* Thunb., and ginger family, can activate PXR and thus inhibit the expression of pro-inflammatory genes downstream of NF-κB (Ren et al., 2019; Yu et al., 2020).

MAPK is an important transmitter of signals from the cell surface to the interior of the nucleus, regulating cell growth, cell differentiation, adaptation to environmental stress, inflammatory response, and many other critical cellular physiopathological processes. MAPK includes p38, JNK, and ERK, which, together with the NF-кB signaling pathway, regulate inflammatory genes’ expression (Figure 2).

#### 2.2.2 TLR4

In recent years, there has been growing evidence that immune system dysregulation, particularly Toll-like receptor (TLR)-mediated innate immune system disorders, is a central player in the pathogenesis of IBD. After activating the TLR signaling pathway, which leads to the induction of many genes that play a role in host defense, including inflammatory cytokines, chemokines, and antigen-presenting molecules (Kordjazy et al., 2018). It has been shown that although TLR activation leads to transcription of inflammatory and immune regulatory genes, TLR signaling in the intestine may also suppress inflammatory responses, thereby maintaining intestinal homeostasis (Shibolet and Podolsky, 2007). In IBD and other intestinal inflammatory diseases, TLR4 is widely upregulated, and TLR4 can act through two signaling pathways, myeloid differentiation factor 88 (MyD88)-dependent and TRIF-dependent (Kawai and Akira, 2007). MyD88 is a junction protein containing the TLR structural domain, which plays a vital role as a downstream signaling factor in the TLR signaling pathway, and TLR4 can be transduced through the MyD88 pathway (Nagpal et al., 2009).

Following receptor-ligand binding, TLRs signaling pathway activation initiates multiple immune responses, with TLR4 inducing type I interferons and inflammatory cytokines. The pathway is activated and propagated through a complex intracellular signaling pathway and *via* a mitogen-activated protein kinase cascade reaction, leading to activation of the transcription factor NF-κB and activator protein 1 (Bhoj and Chen, 2009). In addition, the natural product Panax Notoginseng Saponin can also inhibit the activation of the downstream PI3K/Akt signaling pathway through TLR4 (Lu et al., 2020). Akt plays a vital role in cell survival and apoptosis (Tokuhira et al., 2015), and Panax Notoginseng Saponin can protect the TLR4/AKT signaling pathway by inhibiting rats from Dextran Sulfate Sodium (DSS)-induced intestinal inflammatory injury (Figure 2).

#### 2.2.3 JAK/STAT

The JAK/STAT signaling pathway mainly consists of Janus tyrosine kinase (JAK), tyrosine kinase receptor, Signal Transducer, and Activator of Transcription (STAT). It plays an essential role in cell growth, cell proliferation, cell invasion, cell metastasis, and regulation of apoptotic processes (O’Shea et al., 2013). The current JAK family includes JAK1, JAK2, JAK3, and TYK2, and all are expressed in human cells with close structural similarities. JAK1, JAK2, and TYK2 are widely expressed in various cells, whereas JAK3 is predominantly expressed only in cells of hematopoietic origin.

The STAT family, a group of intracellular proteins that signal and activate transcriptional functions, contains seven members (STAT1-4, 5A, 5B, and 6). STAT3 is most closely associated with immunosuppressive effects and is the only family member whose genetic defect leads to embryonic necrosis (O’Shea et al., 2013). IL-6 is a critical pro-inflammatory factor in the organism and essential signaling for various inflammatory responses, cell differentiation, and proliferative activities. JAK2/STAT3 is an IL-6 downstream signaling pathway, which leads to JAK-mediated STAT3 phosphorylation and regulation of target gene transcription in the nucleus (Eskiler et al., 2019). A specific interaction between NF-κB and STAT3 pathways can jointly promote inflammatory responses. STAT3 and NF-κB synergistically regulate several target genes, associated cytokines, and chemokines. The activation of STAT3 and NF-κB is a result of the combined action of chronic inflammation and the tumor microenvironment (Cho et al., 2011).

In addition, activated JAK can phosphorylate STAT1 and STAT3 and initiate the expression of inflammatory factors IL-6 TNF-α. In contrast, Indigo Naturalis can inhibit STAT1/STAT3 signaling and protect against DSS-induced colitis in mice (Xiao et al., 2019) (Figure 1).

#### 2.2.4 Inflammasome

The inflammasome is a multi-protein complex in the cytoplasm, consisting of receptor protein, junction, and effector molecules. It is a crucial component of the natural human immune system and is involved in various inflammatory responses. According to the receptor protein, the inflammasome includes the NLR family Pyrin domain protein 1 (NOD-like receptor family, pyrin domain containing 1, NLRP1), NLRP3 NLRP4 NLRP6, NLRP7, and NLRP12 (Biasizzo and Kopitar-Jerala, 2020). Secoisolariciresionol Diglucoside is a natural product isolated from Flaxseed, which exerts an immunological role by inhibiting NLRP1 from alleviating DSS-induced colitis in mice (Wang et al., 2020).

**FIGURE 3:**
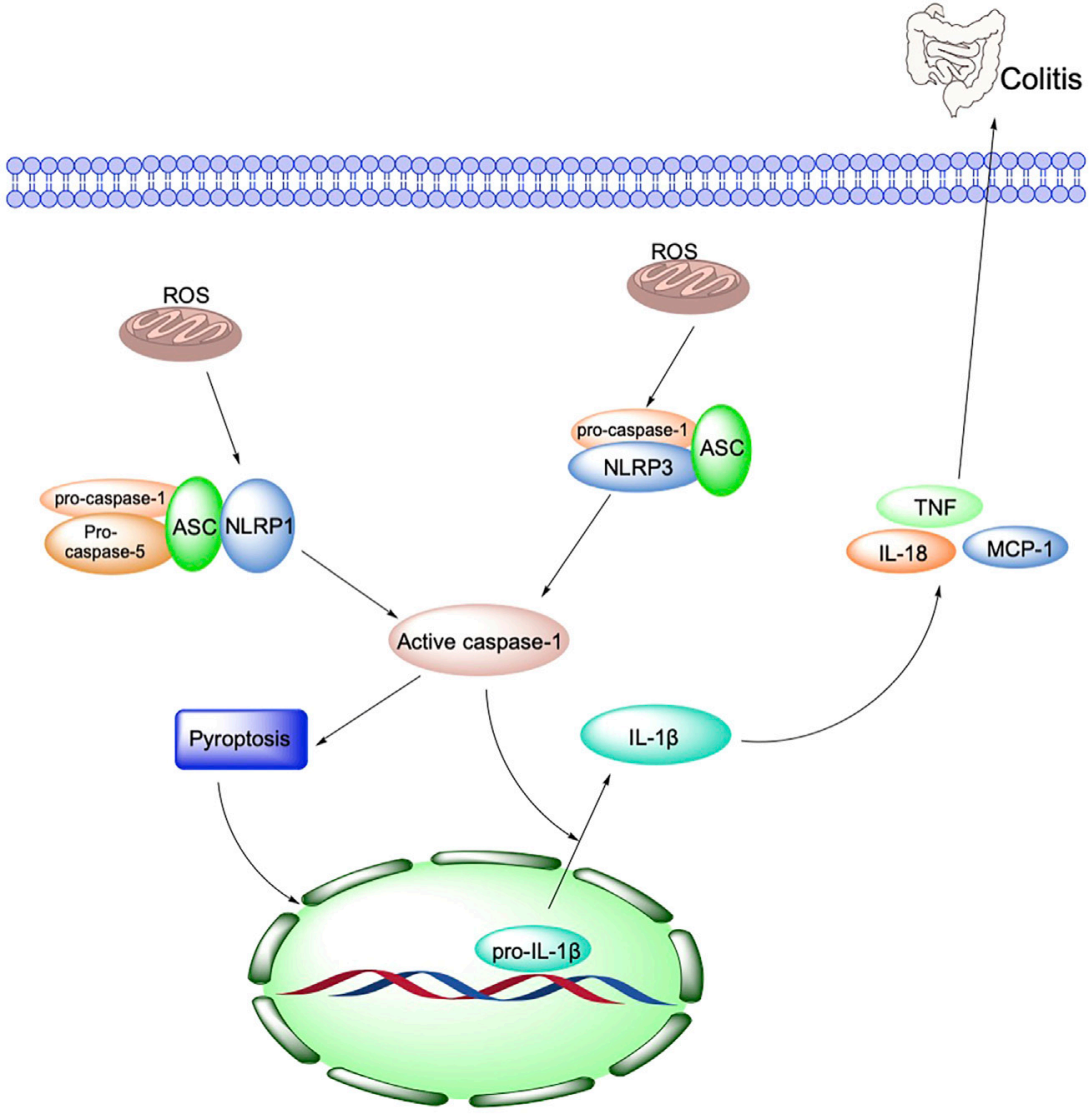
Oxidative stress and NLRP3 are key mechanisms in the pathogenesis of IBD.

NLRP3, the most widely studied inflammasome, is a high molecular mass protein complex composed of NLRP3 protein, Apoptosis-associated Speck-like protein Containing a CARD (ASC), and cysteine aspartate protease 1 (caspase-1). NLRP3 is an essential factor in the host defense response. It is capable of modulating intrinsic immunity, and various environmental stimulants, multiple microorganisms, and endogenous or exogenous danger signals that can activate NLRP3 inflammatory vesicles to release cytokines, induce caspase-1-dependent programmed cell death, and participate in a variety of inflammatory disease processes (Biasizzo and Kopitar-Jerala, 2020) (Figure 3).

#### 2.2.5 PPAR

The peroxisome proliferator-activated receptor (PPAR) is a nuclear hormone receptor superfamily that regulates numerous gene expressions. PPARs include three isoforms: PPAR-α, PPAR-γ, and PPAR-β/δ, which have different tissue distribution and functions. Among them, PPAR-γ is the most widely studied isoform, widely distributed in several tissues and organs, including the intestine, and regulates lipid metabolism, inflammatory response, cell proliferation, and fibrosis (Decara et al., 2020) (Figure 2).

### 2.3 Gut Microbes and Metabolomics

Gut microbes maintain a symbiotic relationship with their hosts and have essential functions such as regulating host metabolism, immunity, and intestinal barrier function. Under physiological conditions, intestinal microorganisms promote the digestion of host dietary fiber and provide beneficial active metabolites such as short-chain fatty acids (SCFAs) and vitamins for the body. Besides, it maintains a dynamic balance with the host immune system, tolerates the presence of normal intestinal bacteria, and inhibits the proliferation and expansion of pathogenic microorganisms (Zhang et al., 2015). Disturbances in the intestinal flora can lead to an over-activation of the host immune system, which induces an inflammatory state, and both intestinal inflammation and IBD are associated with intestinal flora dysbiosis (Shen et al., 2018). In addition, ecological dysbiosis increases the proportion of harmful bacteria in the intestine and releases enterotoxins that increase intestinal permeability, which induces the production of immunosuppressive proteins, leads to immune dysfunction, destroys intestinal epithelial cells, and affects energy metabolism leading to intestinal inflammation (Lobionda et al., 2019).

Metabolomics allows qualitative and quantitative analysis of all small molecule metabolites in an organism and searches for the relationship between metabolites and physiopathological changes. Gut microbial and host co-metabolites include two types of substances: those co-metabolized by the host and gut flora and those metabolized by the host in the gut flora. The detection of dynamic changes in the intestinal flora and host co-metabolites by metabolomics demonstrates the metabolic status of intestinal flora in the host. It can provide clues and directions for studies such as the mechanism of drug therapy for IBD (Hamer et al., 2008). The main intestinal flora included Firmicutes, Bacteroidetes, Epilonbacteracota, Proteobacteria, Deferribacteres, Patescibacteria, Tenericutes, and Actinobacteria 8 different *Bacteroides*, with Firmicutes and Bacteroidetes accounting for the highest percentage. The ratio of Firmicutes to Bacteroidetes was significantly lower in mice treated with DSS, while the natural products were 2,3,5,4′-Tetrahydroxystilbene 2-O-β-D-glucoside and *Persea americana* Mill. Ethanol extracts increased the ratio and improved gut microbiota homeostasis disrupted by DSS (Ma X. et al., 2018; He et al., 2021).

**FIGURE 4:**
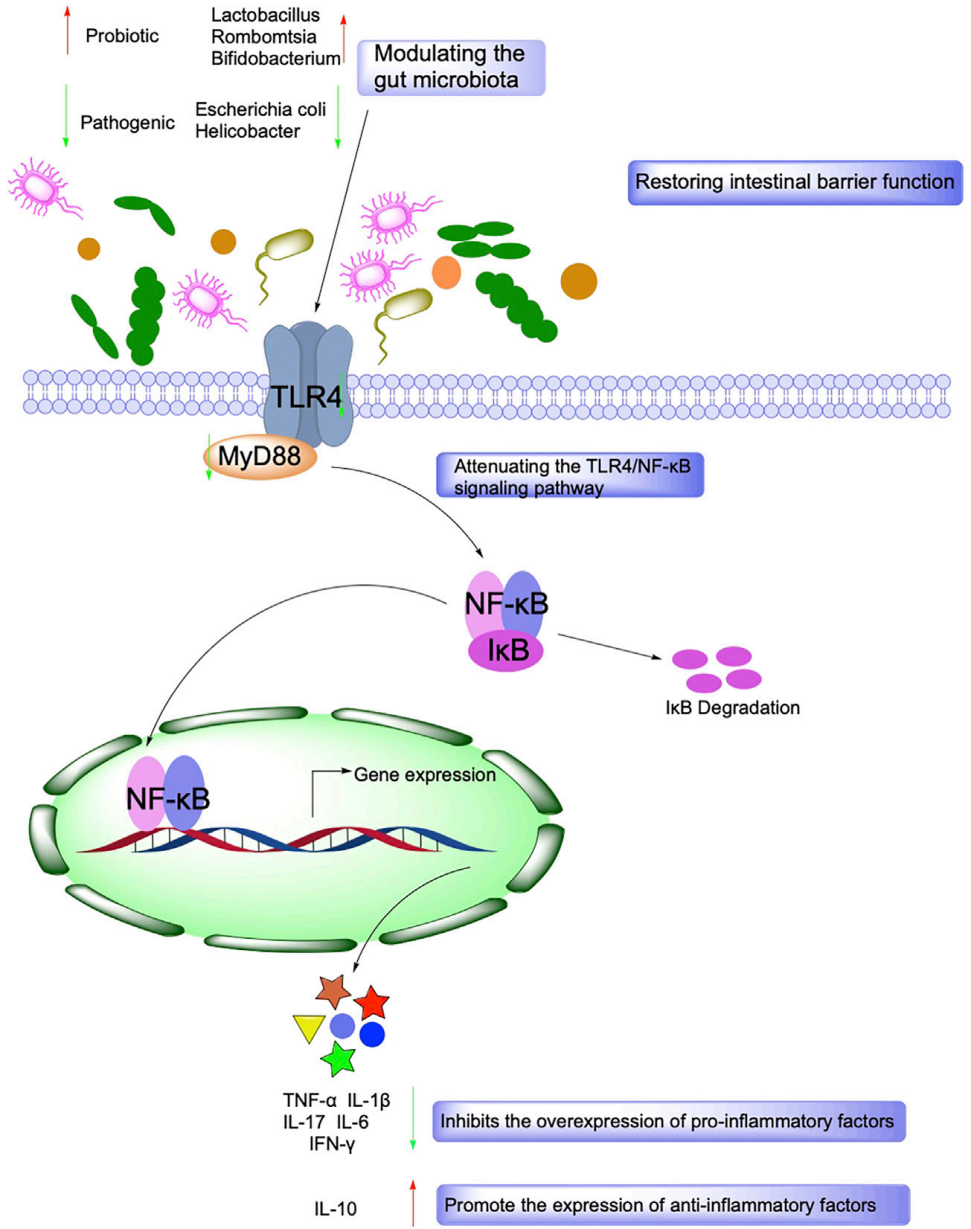
Mechanisms associated with the role of Intestinal barrier function and gut microbiota in IBD.

SCFAs, including formic acid, acetic acid, propionic acid, isobutyric acid, butyric acid, isovaleric acid, and valeric acid, are rapidly absorbed by the hindgut and store both energy and reduce osmotic pressure. SCFAs are essential for maintaining the normal function of the large intestine and the morphology and function of colonic epithelial cells. SCFAs also promote sodium absorption, and butyric acid increases the production of beneficial bacteria *Lactobacillus* and reduces the production of harmful bacteria *E. coli* (Su et al., 2020) (Figure 4).

### 2.4 Intestinal Mucosal Mechanical Barrier

The intestinal mucosal epithelial cells and the tight junction (TJ) between the cells together constitute the intestinal mucosal mechanical barrier. The integrity of the intestinal mucosal mechanical barrier is jointly determined by the composition and function of the epithelium and TJ. The sealing properties of the intestinal epithelium are achieved by a monolayer of columnar epithelial cells, which form a moderately tight monolayer through the intercellular TJ (Epple and Zeitz, 2012). TJ can create selective osmotic closure between adjacent epithelial cells and delineates the boundary between the apical and basolateral membrane domains. Disruption of TJ infiltrates harmful luminal molecules and causes disturbances in the immune system of the intestinal mucosa and inflammation. Thus, the TJ can act as a trigger for the development of intestinal and systemic diseases (Lee, 2015). The TJ comprises the cytoplasmic protein ZO (Zonula Occludens Proteins) family, transmembrane proteins (Tricellulin, Nectin, Junctional Adhesion Molecules, Occludin, and Claudins), and cytoskeletal structures. Occludin and Claudins can modulate the function of the intestinal mucosal mechanical barrier by affecting TJ permeability. In addition to regulating intestinal mucosal TJ protein expression, one important pathway is the activation of myosin light-chain kinase (MLCK), which causes myosin light-chain phosphorylation, further causing cytoskeletal contraction and thus altering the intestinal mucosa’s mechanical barrier structure and function (Puthia et al., 2006). MLCK is a Ca^2+^/CaM-regulated protein kinase that regulates the permeability of tight junction proteins and is a crucial enzyme in cell contraction, mediating the contraction of smooth and non-smooth muscle, thereby opening the intercellular gap and increasing the permeability of the cell barrier. Phosphorylation of the myosin light chain increases the cell gap and triggers endocytosis of tight junction proteins, thus further affecting tight junctions.

The primary pathological manifestation of impaired intestinal mucosal barrier function is altered intestinal mucosal permeability, apoptosis of intestinal epithelial cells (IECs), and disruption of intercellular TJs are the main relevant factors. In the pathogenesis of IBD, inflammatory factors such as TNF-α and INF-γ induce the expression of apoptosis-related proteins like caspase-1 in IECs, which in turn inhibit the expression of anti-apoptotic proteins such as Bcl-2, inducing apoptosis in a large number of IECs(Koshiji et al., 2020).

### 2.5 Antioxidant

It has been shown that the pathogenesis of IBD is associated with the excessive production of reactive oxygen species (ROS) and the disruption of the antioxidant system. ROS are essential signaling molecules in the body, and activation of neutrophils and macrophages can contribute to ROS overproduction, which triggers a series of chain reactions (Iborra et al., 2011). Excess ROS can lead to oxidative cellular damage and activate inflammatory factors in the intestine to trigger an inflammatory response, affecting the expression of multiple inflammatory signaling pathways and related effector proteins, which aggravates tissue damage and accelerates the progression of IBD (Ma J. et al., 2018). The severity of IBD is also related to the level of superoxide dismutase (SOD), an essential antioxidant enzyme in the body, which is the primary substance for scavenging oxidative free radicals, and its disproportionation is the most crucial role in cellular defense against oxidative stress. It was shown that in DSS-induced UC mice, Root Extract of *Polygonum cuspidatum* Siebold & Zucc. was able to increase SOD, Catalase (CAT), and Glutathione peroxidase (GSH) and decrease the activity of malondialdehyde (MDA) as an antioxidant (Liu B. et al., 2018). Not only that, but Ursolic acid also decreased MDA content in DSS-induced mice, increased SOD activity in colonic homogenates, and exerted antioxidant and anti-inflammatory properties by inhibiting the activation of the NF-κB pathway (Liu et al., 2016).

When phagocytes in the surrounding tissues and body fluids are activated, myeloperoxidase (MPO) can be secreted into the extracellular environment and phagocytic vesicles to exert pathogenic bacterial killing effects through the production of hydrogen peroxide and chloride as substrates. Not only that, MPO is produced in excess while killing pathogenic bacteria, causing inflammatory tissue damage (Galijasevic, 2019). The reactivity of hydrogen peroxide is enhanced by making hypochlorous acid, free radicals, and reactive nitrogen species. The interaction of nitric oxide with oxygen or oxygen-related reaction intermediates (e.g., superoxide) can produce large amounts of ROS (Neri et al., 2017).

On the other hand, natural products can also mitigate oxidative responses by regulating signaling pathways associated with oxidative stress. The current study found that Licochalcone A and Rutaecarpine are involved in the regulation of NF-κB, Nuclear factor E2-related factor 2 (Nrf2), and other pathways. Among them, the Nrf2 pathway mainly stimulates the expression of SOD and GPx genes. Natural products can upregulate the relative expression of antioxidant enzyme-related factors in the colonic tissues of UC mice and increase the activity of antioxidant enzymes (SOD, GSH-Px, CAT) in serum by activating the Nrf2 pathway (Liu D. et al., 2018; Zhang et al., 2020).

### 2.6 Autophagy

Autophagy regulates cells by autophagy-related genes (ATG) that allow intracellular aged and damaged cellular material to be delivered to lysosomes for degradation. The ability of autophagy to selectively target intracellular pathogens for destruction is now considered a vital aspect of the innate immune response (Levine and Kroemer, 2019). As an essential process for maintaining homeostasis in the intestine, autophagy dysfunction plays a vital role in IBD development, and cellular autophagy is often depressed in IBD patients. However, the exact mechanism of action remains to be elucidated. It has been found that autophagy plays a crucial role in maintaining intestinal barrier and mucosal homeostasis, and abnormal ATG can lead to defective autophagy, which impairs the critical role of autophagy in limiting the inflammatory cytokine response, causing damage to the intestinal epithelial barrier and uncontrolled intestinal inflammation (Lassen and Xavier, 2018).

### 2.7 Angiogenesis

Angiogenesis is the process of forming new blood vessels by outgrowth or other means based on existing capillaries. Typically, angiogenesis is an essential part of the growth and development process under normal physiological conditions. It has been found that the overexpression of vascular endothelial growth factor-A (VEGF-A) in colitis mice increases angiogenesis in the intestinal mucosa and stimulates leukocyte adhesion, worsening the condition of mice, suggesting that IBD is accompanied by angiogenesis (Scaldaferri et al., 2009). The growth and inhibition of blood vessels in the body are in a dynamic balance under normal conditions to maintain a relatively stable state. However, once imbalanced, it can lead to the development of many diseases. In the early stages of IBD, serum levels of pro-angiogenic factors are elevated and imbalanced with the action of angiogenic inhibitors, thus leading to pathological angiogenesis. The immune-inflammatory response and angiogenesis in IBD are mutually reinforcing processes, the dysfunctional immune response being one of the causative factors of IBD (Pousa et al., 2008). Many factors in inflammatory tissues can regulate angiogenesis. Hypoxia is a significant angiogenic stimulus, as vascular smooth muscle cells proliferate and produce hypoxia-inducible factor-1α (HIF-1α) in large quantities under hypoxic conditions. VEGF is the most direct vascular endothelial cell pro-divider, and VEGF binds to its receptor and exerts a series of angiogenic-promoting biological functions. In addition, there are many inflammatory cells (such as macrophages, mast cells, lymphocytes, and fibroblasts) in inflammatory lesions, which can directly or indirectly release a large number of pro-angiogenic factors to promote angiogenesis. These include the VEGF family and its receptors, integrins αvβ3 and αvβ5, monocyte chemotactic protein (MCP), platelet-derived growth factor-BB (PDGF-BB), matrix metalloproteinase (MMP), and TGF-β1. It has been shown that angiogenesis-promoting and inhibitory factors can be stored within distinct platelet alpha granules. During this period, angiogenesis is involved by selectively releasing different kinds of angiogenic regulators within platelet alpha granules. This can actively mobilize various membrane surface receptors to participate in self-adhesion, aggregation, and angiogenesis (Eder et al., 2015).

## 3 TCM and IBD

Table 1 shows that TCM has made a series of advances in IBD treatment. Indirubin, the active ingredient of a Chinese medicine formulation, significantly inhibited the infiltration of CD4^+^ T cells in the colon of DSS-induced UC mice (Gao W. et al., 2016). *Tripterygium wilfordii* polyglycosides can modulate Th17/Treg imbalance in IBD patients by inhibiting Th17 cell differentiation, promoting Tregs differentiation, and modulating inflammatory cytokines by enhancing the *in-situ* levels of Foxp3^+^ Tregs, which in turn reduces microscopic inflammation (Li et al., 2014). In 2,4,6-trinitrobenzene sulfonic acid (TNBS)-induced colitis mice, Treg levels were significantly increased by *Astragalus aaronii (Eig)* Zohary polysaccharide (Zhao et al., 2016). Oxymatrine extracted from the roots of *Sophora flavescens* Aiton, the ingredient edible bird’s nest and *Tripterygium wilfordii* polyglycoside were able to regulate Th17/Treg balance and have a protective effect against colitis (Wang Y. et al., 2019; Song et al., 2019; Fan et al., 2021; Zhang et al., 2021). In addition, botanical drugs *Portulaca oleracea* L., Ripened Pu-erh tea, and *Fagopyrum cymosum* (Trevir.) Meisn., *Citrus aurantium* L., and Mu Dan Pi have been reported to inhibit the protein expression of NF-κB p65 in mice with colitis, showing significant anti-inflammatory effects (Yang et al., 2016; Ge et al., 2017; Wang Z. et al., 2018; He et al., 2018; Huang Y. et al., 2020; Chen et al., 2020). Wu-Mei-Wan has beneficial effects on IBD and the possible mechanism may involve blocking the IL-6/JAK2/STAT3 signaling pathway, providing a promising new therapy for the treatment of IBD (Wu F. et al., 2020). In past studies, Ripened Pu-erh Tea, extracts of *Abelmoschus manihot var. manihot,* and *Portulaca amilis* Speg. have been shown to alleviate colitis by modulating the inflammatory response and increasing the levels of PPAR-γ (Kong et al., 2018; Zhang W. et al., 2019; Huang et al., 2021). Not only that, Barley Leaf improves intestinal mucosal barrier function by activating PPAR-γ signaling (Li D. T. et al., 2021). TCM such as *Lonicera japonica* Thunb. polysaccharide, *Bruguiera gymnorhiza* (L.) Lam., and Red Yeast Rice can increase the abundance of probiotic bacteria back to normal levels (Huang YP. et al., 2020; Lin et al., 2020; Zhou et al., 2021). Nevertheless, the Chinese medicine *Panax notoginseng* (Burkill) F.H.Chen can repair vascular damage in experimental colitis by reducing the concentration of MPO to reduce inflammation and oxidative stress in the colonic mucosa (Wang SY. et al., 2017).

**TABLE 1:** The targets of TCM in attenuating IBD.

TCM	Experiment models	Targets	References
Cinobufacini	DSS-induced Mice	reduced M1 macrophages and increased M2 macrophages	Wang et al. (2019a)
Indirubin	DSS-induced Mice	inhibited the infiltration of CD4^+^T Lymphocytes	Gao et al. (2016a)
edible bird’s nest	DSS-induced Mice	Th17/Treg	Fan et al. (2021)
Tripterygium wilfordii polyglycoside	TNBS-induced Rats	Th17/Treg	Li et al., 2014, Zhang et al., 2021
*Astragalus* polysaccharide	TNBS-induced Mice	Treg cells	Zhao et al. (2016)
Oxymatrine	DSS-induced Mice	Th17/Treg	Wang et al. (2019b)
Clematichinenoside AR	IL-10^−/−^ mice	Th17/Treg	Song et al. (2019)
Portulaca oleracea L	DSS-induced Mice	NF-κB	Yang et al., 2016, Wang et al., 2018b
Ripened Pu-erh tea	DSS-induced Mice	NF-κB	Huang et al. (2020a)
Fagopyrum cymosum	TNBS-induced Mice	NF-κB	Ge et al. (2017)
Citrus aurantium L	TNBS-induced Rats	NF-κB	He et al. (2018)
Mu Dan Pi	DSS-induced Mice	NF-κB	Chen et al. (2020)
Wu-Mei-Wan	TNBS-induced Mice	IL-6/JAK2/STAT3	Wu et al. (2020a)
Ripened Pu-erh Tea	DSS-induced Mice	PPAR-γ	Kong et al. (2018)
Flos Abelmoschus manihot	DSS-induced Mice	PPAR-γ and Th17/Treg	Zhang et al. (2019c)
Portulaca	DSS-induced Mice	PPAR-γ	Huang et al. (2021)
Taraxasterol	DSS-induced Mice	PPAR-γ	Chen et al. (2020)
Barley Leaf	DSS-induced Mice	PPAR-γ	Li et al. (2021b)
*Lonicera japonica* Thunb polysaccharide	DSS-induced Mice	Intestinal Microbiota	Zhou et al. (2021)
*Bruguiera* gymnorrhiza (L.) Lam. Fruit	DSS-induced Mice	Intestinal Microbiota	Lin et al. (2020)
Sulforaphene	TNBS-induced Rats	Intestinal Microbiota	Li et al. (2019)
Red Yeast Rice	Winter4-induced Mice	Intestinal Microbiota	Huang et al. (2020b)
Panax notoginseng	DSS and IA-induced Rats	Antioxidant and MPO	Wang et al. (2017b)

## 4 Natural Products and IBD

As shown in Table 2, natural products have made advances in IBD treatment.

**TABLE 2:** The targets of Natural Products in attenuating IBD.

Categories	Natural product	Experiment models	Targets	References
Polysaccharide	*Astragalus membranaceus* polysaccharides	RSL3-induced Caco-2 cells	NF-κB signaling; reduce NO, MDA, TNF-α and IL-6, increase TGF-β1, SOD and GSH activities; downregulate the expression of NLRP3, caspase-1 and ASC in colonic tissues, thus preventing the activation of NLRP3, reduces the expression of IL-18, IL-1; reduce PTGS2, FTH, and FTL, Nrf2/HO-1 pathway	Gao et al. (2016b), Lv et al. (2017), Ko et al. (2005), Tian et al. (2017), Chen et al. (2021)
		DNBS/TNBS-induced Rats		
		DSS-induced mice		
	*Dendrobium officinale* polysaccharides	DSS-induced mice	activate GPRs, upregulate IL-10 and decreases caspase-1, IL-6, TNF-α, IFN-γ, IL-18, IL-1β; downregulate β-arrestin 1, block NLRP3; increase Nrf2, Keap1, HO-1, and NQO-1, inhibit ROS production and MDA, increase SOD and GSH; improve the diversity of the intestinal microbiota, stimulate SCFAs in the colon, increase acetate, butyrate, ZO-1, ocludin, and decreasing the permeability of the intestinal epithelium function	Liang et al. (2018a), Wen et al. (2021), Liang et al. (2019)
	*Lycium barbarum* polysaccharide	LPS-induced RAW264.7 cells	inhibit the TLR4/NF-κB pathway, reduce IL-6 and TNF-α, suppresse NO and iNOS	Peng et al. (2014)
		DSS-induced Mice		
	*Schisandra chinensis* polysaccharide	DSS-induced Mice	regulate IFN-γ, TNF-α, IL-1β, IL-17, IL-13, IL-6, improve the diversity and composition of the intestinal microbiota, increase SCFAs levels	Su et al. (2020)
	*Atractylodes macrocephala* polysaccharide	DSS-induced Mice	modulate the ability of the intestinal microbiota to produce SCFAs, intestinal microbes, digest food nutrients, amino acids and bile acid metabolism	Feng et al. (2020)
	*Lonicera japonica* polysaccharide	DSS-induced Mice	promote NK and CTL, attenuate the apoptosis of splenic lymphocytes, restore the diversity of intestinal flora	Zhou et al. (2021)
Saponins	Baicalin	TNBS-induced Rats	increase CAT, SOD and GSH, block NF-κB and PI3K/Akt signaling pathways, reduce IL-6, IL-1β and TNF-α, increasing IL-10, reduce oxidative stress damage; inhibit MIF, regulate macrophage function, promote CD4^+^CD29^+^ cells, regulate Th17/Treg balance; upregulate IRF4, induce the differentiation of macrophages into M2-type macrophages with anti-inflammatory effects; downregulate caspase-3, caspase-9, Bax and FasL, promote Bcl-2, and effectively inhibit apoptosis; downregulate miR-191a, increase occludin, ZO-1, and MUC-2, and reduce IEC-6; reduce the ratio of Firmicutes to Bacteroidetes, increase butyric acid, and regulate the metabolism of SCFAs	Shen et al. (2019), Zhu et al. (2020a), Shen et al. (2019), Zhu et al. (2020a), Dai et al. (2012), Zhu et al. (2016), Zhu et al. (2020b), Zhu et al. (2020a), Wang et al. (2017a), Zhu et al. (2020)
		DSS-induced mice		
		LPS-induced mouse peritoneal macrophages		
		LPS-induced		
		RAW264.7 cells		
	Naringin	AA/TNBS-induced Rats	up-regulate SOD and GSH, reduce MDA and MPO; prevent LDH and ALP by decrease XO and ALP, restore oxidative homeostasis, reduce DNA damage; reduce the abundance of pathogenic bacteria; activate PPARγ, inhibit NF-κB, MAPK pathway, and NLRP3, regulate the expression of ZO-1	Cao et al. (2018), Hambardikar and Mandlik (2022), Kumar et al. (2014), Cao et al. (2021), Cao et al. (2018)
		DSS-induced Mice		
	Paeoniflorin	LPS-induced Caco-2 cells	inhibit DCs by JAK/STAT signaling pathway, decreaseIL-12 and the percentage of MHC-II^+^CD86^+^ DCs maturation, restoreDC-mediated Th17/Treg homeostasis, reduce IL-17 secretion, upregulate Foxp3, IL-10, induce the differentiation of naive T cells into CD4^+^CD29^+^ Treg cells; reduce CCL11, CCL24, CCL26, eosinophil migration in the intestine; block MDP/NOD2 pathway, inhibit NF-κB p65, increase cupped cells, restore the crypt structure, reduce the infiltration area of pathogenic bacteria; restore TJ proteins, activate Nrf2/HO-1	Zheng et al. (2020), Li et al. (2021c), Luo et al. (2021b), Wu et al. (2019)
		TNBS-Mice		
		DSS-induced Mice		
	Ginsenosides Rg1	mouse bone marrow macrophages	block PTEN and SOCS, inhibit PI3K/Akt and STAT, regulate Tfh/Treg cell; target and regulate Nogo-B/RhoA, regulate M1 and M2 macrophages, promote NLRP12, suppresses IL-1β and TNF-α through TLR4; improve the diversity of colonic microbiota	Jin et al. (2021), Zhu et al. (2017), Long et al. (2022), Long et al. (2022)
		DCs		
		DSS-induced Mice		
	*Panax notoginseng* saponin	Pam3CSK4-induced RAW 264.7 cells	decrease the phosphorylation of PI3K and Akt, inhibit PI3K/Akt, increase IL-10, decrease the percentage of CD11BF4/80-labeled macrophages, induce macrophage polarization to anti-inflammatory CD206^+^ M2 macrophages; inhibit NO release, target the p38 MAPK and TLR/NF-κB; increase Bcl-2 and Bcl-3, downregulate caspase-3 and Bax; increase ZO-1, claudin-1 and ocdcludin	Lu et al. (2020), Luo et al. (2021a), Lu et al. (2020), Lu et al. (2020)
		DSS-induced Rats		
		DSS-induced Mice		
	Astragaloside IV	LPS-induced CCD-18Co cells mouse bone marrow macrophages	reduce inflammatory factors, downregulate NF-κB; regulate STAT3, inhibit STAT1 activation, increase M2 macrophage markers CD206, Ym1, and TGF-β, inhibit pro-inflammatory M1 macrophage markers iNOS, IL-6, and IL-1β activity, decrease M1 and M2 ratios; increase ATP content, stimulate nuclear translocation of β-Catenin, accelerate epithelial cell proliferation, inhibit claudin-5 and ocdcludin	Wu and Chen (2019), Tian et al. (2021), Jiang et al. (2017)
			TNBS-induced Rats	
			DSS-induced Mice	
Alkaloi Jing et al., 2018ds	Matrine	TNBS-induced Mice	activate Nrf2, suppress JAK2/STAT3, down-regulate inflammatory factors, regulate apoptosis-related factors epithelial cell apoptosis; increase TJs protein, increase mucin-producing cells and MUC-2, involve the activation of the PPAR-α; promote Treg differentiation by blocking RhoA/ROCK, down-regulate the ratio of Th1 to Th17; increase GSH and SOD, inhibit MPO, iNOS, COX, and ROS; block the PI3K/Akt signaling pathway, prevent Bcl-2 and Bad, up-regulate of caspase-3 and caspase-9; block the TLR-9/MyD88/NF-κB pathway, restore TJs protein	Fang et al. (2018), Yao et al. (2021), Chen et al. (2022a), Li et al. (2019), Wang et al. (2019b), Tang et al. (2020), Chen et al. (2017), Li et al. (2022)
		DSS-induced Mice		
		DSS-induced NCM460 cells		
		TNBS-induced Rats		
	Berberine	DSS-induced Mice	increase sIgA; down-regulate STAT1 and STAT3 phosphorylation, inhibit the NF-κB signaling pathway, decrease the ratio of Th1 and Th17; activate PKB/SOCS1, inhibit the phosphorylation of p65, reduce M1 macrophages, and regulates the ratio of M1 and M2 type macrophages; activate AhR, promote TJs epithelium; restore carbohydrate digestion and absorption, glycolysis, gluconeogenesis and amino acid metabolism, decrease the proportion of harmful bacteria; activate the Nrf2 pathway and induce P-gp	Li et al. (201), Liu et al. (2018c), Jing et al. (2021), Liao et al. (2020), Jing et al. (2018)
		DSS-induced Rats		
		TNBS-induced Mice		
		RAW264.7 cells		
		Caco-2 cells		
	Piperine	SW480 cells	block MAPK, inhibit CXCL8; inhibit ILs, TNF-α, COX-2 and iNOS via blocking the IκB-α/NF-κB, down-regulate caspase-1, inhibit apoptosis, ameliorate TJs; induce CYP3A4 gene expression of CYP450 enzyme lineage	Hou et al. (2015), Guo et al. (2020), Hu et al. (2015)
		HT-29 cells		
		LS174T cells		
		DSS-induced Mice		
		TNBS-induced Rats		
	Sinomenine	DSS-induced Mice	inhibit IL-1β, TNF-α, downregulate miRNA-155 and other related inflammatory cytokines	Yu et al. (2013)
Organic Acids	Chlorogenic acid	DSS-induced Mice	down-regulate MPO by inhibiting TLR4-mediated PI3K/Akt and NF-κB pathways; increase SOD1 and CAT, decrease MDA and ROS; attenuate apoptosis by inhibiting the expression of HO-1, Bax, caspase-8 and caspase-9; improve intestinal flora by regulating amino acid and lipid metabolism; promote SCFA production, increase butyric acid and reduce mucosal damage	Zhang et al. (2019b), Chen et al. (2022b), Wan et al. (2021), Vukelic et al. (2018), Xie et al. (2021)
		High Fat Diet Rats		
		LPS-induced RAW264.7 cells		
	Gallic acid	DSS/TNBS induced Mice	inhibit p-IκBα and p-NF-κB, increase IL-4 and IL-10, down-regulate IL-6, IL-12, IL-17, IL 23, TGF-β and TNF-α, up-regulate Nrf2, UDP-GT and NQO1; reduce MPO, iNOS and COX-2, improve mucosal damage	Pandurangan et al. (2015a), Pandurangan et al. (2015b)
	Ursolic acid	DSS-induced Mice	inhibit NF-κB and MAPK, reduce TNF-α, IL-1β, COX-2, and iNOS; inhibit JAK/STAT activation and JNK signaling, up-regulate CAT and T-SOD, reduce ROS production; reduce intestinal bacterial community abundance, regulate fatty acid metabolism, and affected immune cell infiltration and cytokine expression, which may be related to MAPK, IL-6/STAT3, AMPK/FOXO and PI3K signaling pathways	Chun et al. (2014), Jang et al. (2014), Liu et al. (2016), Wei et al. (2022), Sheng et al. (2021)
		DSS/TNBS-induced Mice		
		SDS-induced *Drosophila*		
		LPS-induced mouse peritoneal macrophages		
	Rhein	LPS-induced RAW264.7 cells	down-regulate NF-κB and NLRP3, activates the Nrf2/HO-1/NQO1 pathway, inhibit NOX2 subunit expression and translocation, down-regulate protein expression levels of IL-6, IL-1β, TNF-α, iNOS, and COX-2, reduce NO production, increase beneficial bacteria, decrease pathogenic bacteria, improve dysbiosis, inhibit of PI3K/Akt/mTOR; regulate uric acid metabolism, restore the barrier function, decrease intestinal permeability, increase claudin-1, E-cadherin and the secretion of mucus	Dong et al. (2022), Wu et al. (2020b)
		DSS-induced Mice		
Flavonoids	Luteolin	TNF-α and IFN-γ-induced Caco-2 cells	increase SOD and CAT, down-regulate MDA, activate the Nrf2 signaling pathway, inhibit p-STAT1, p-JAK1 expression, block NF-κB pathway transduction, reduce COX-2, iNOS, IL-8, NO; block the STAT3 signaling pathway, increase ZO-1, claudin-1, and OCLN; inhibit MEK and ERK phosphorylation, decrease 5-HT and TPH-1; alter the diversity and composition of the gut microbiota, DNA repair, ribosome, purine and pyrimidine metabolism	Nunes et al. (2017), Li et al. (2016), Suga et al. (2021), Li et al. (2021a)
		DSS-induced Mice		
		TNBS-induced Mice		
	Cardamonin	LPS-induced RAW 264.7 cells, THP-1 cells mouse bone marrow macrophages	inhibit the up-regulation of TLR4 and MyD88, reduce TNF-α and IL-6; activate AhR, promote the Nrf2/NQO1 signaling pathway, and inhibit NLRP3; reduce caspase-3, MPO, iNOS, COX-2 and MDA, reduce oxidative stress	Ren et al. (2015), Wang et al. (2018a), Ali et al. (2017)
		AA-induced Rats		
	Myricetin	DSS-induced Mice	increase the ratio of CD4^+^CD29^+^ Treg, restore Th17/Treg balance; reduce MPO and MDA, decrease NO, increase SOD and GSH; increase claudin-1 and occludin, improve the intestinal flora, increase the metabolism of ascorbic acid, aldehyde and lipids	Qu et al. (2020), Zhao et al. (2013a), Qu et al. (2020), Miao et al. (2021)
Terpenoids	Tripterygium wilfordii Hook F	DSS-induced Mice	regulate Th17/Treg imbalance; enhance the *in situ* levels of Foxp3^+^Tregs, reduce microscopic inflammation; inhibit NOXs-ROS-NLRP3 signaling pathway antioxidant and anti-lipid oxidation, suppress the expression of pro-inflammatory factors; corticosteroid-like functions	Ogino et al. (2013), Li et al. (2014), Fangxiao et al. (2019), Chen et al. (2005)
	Andrographolide	LPS-induced RAW264.7 cells	activate AMPK, block NF-κB and p38 MAPK, reduce NO production, decrease iNOS and COX-2; regulate STAT3 signaling pathway, decrease IL-23, IL-17 and IFN-γ; block the IL-4R/STAT6 signaling pathway, reduce the specific binding of IL-4/IL-13 to IL-4R, inhibit MPO activity and TNF-α secretion	Kim et al. (2019), Zhu et al. (2018), Zhang et al. (2019a)
		DSS-induced Mice		
		OXA-induced Rats		

### 4.1 Polysaccharide

Polysaccharide is one of the four essential substances that constitute life which is widely found in plants, animals, microorganisms, lichens, and seaweeds. Polysaccharides play biologically active roles in anti-tumor, anti-inflammatory, anti-viral, hypoglycemic, anti-aging, anti-coagulation, and immune promotion. Therefore, the research and development of polysaccharides have attracted more and more attention (Zeng et al., 2019).

#### 4.1.1 *Astragalus membranaceus* Polysaccharides

*Astragalus membranaceus* polysaccharides (APS) are the main components of *Astragalus aaronii* (Eig) Zohary, functioning as immune promoters or modulators (Jin et al., 2014). APS promotes the transformation of T cells to a Th2 anti-inflammatory cell phenotype by inhibiting NF-κB signaling (Gao YJ. et al., 2016; Lv et al., 2017). APS can modulate TLR/NF-κB signaling pathway, significantly reduce NO, MDA, TNF-α, and IL-6 in UC mice, and increase TGF-β1 levels and SOD and GSH activities. Besides, it inhibits inflammatory responses in mice, activates the repair effect of their tissues against oxidative stress damage, and alleviates the pathological state of inflammation (Ko et al., 2005). In addition, APS can also downregulate the expression of NLRP3, caspase-1, and ASC in colonic tissues, thus preventing the activation of NLRP3, which in turn reduces the expression of IL-18 and IL-1β and alleviates the DSS-induced colonic inflammatory response (Tian et al., 2017). It has been shown that APS can reduce the expression of iron apoptosis-related gene expressions such as Prostaglandin endoperoxide synthase-2, Ferritin heavy chain, and Ferritin light chain. Iron apoptosis is prevented, which may be related to regulating the Nrf2/HO-1 pathway (Chen et al., 2021).

#### 4.1.2 *Dendrobium officinale* Polysaccharides

*Dendrobium officinale* polysaccharides (DOPS) are polysaccharide components of the *Dendrobium catenatum* Lindl. and with broad pharmacological activity (Liang et al., 2018b). Treatment with DOPS activates G protein-coupled receptors, upregulates the expression level of IL-10, and decreases the levels of caspase-1, IL-6, TNF-α, IFN-γ, IL-18, and IL-1β. DOPS can downregulate the activity level of β-inhibitory protein 1activity levels, block the NLRP3 signaling pathway, and significantly attenuate the DSS-induced colitis response (Liang et al., 2018a). By downregulating the TNF-α signaling pathway, DOPS can increase the mRNA and protein expression of Nrf2, Keap1, HO-1, and NQO-1, inhibit ROS production and MDA activity, and increase the activity of antioxidant enzymes SOD and GSH in mouse colonic tissues, significantly improving antioxidant activity (Wen et al., 2021). In addition, DOPS can enhance the diversity of the intestinal microbiota in mice with colitis by upregulating the ratio of Bacteroidetes, *Lactobacillus*, and rumenococci, while reducing the abundance of Aspergillus to some extent and restoring the intestinal barrier by stimulating the production of SCFAs in the colon, increasing the levels of acetate and butyrate and the expression of ZO-1, occludin, and decreasing the permeability of the intestinal epithelium function (Liang et al., 2019).

#### 4.1.3 Others

In the LPS-stimulated inflammation model of RAW 264.7 cells, *Lycium barbarum* polysaccharide exerts anti-inflammatory and antioxidant effects by inhibiting the TLR4/NF-κB pathway, reducing the expression levels of IL-6 and TNF-α, and suppressing the overproduction of NO and the mRNA expression of iNOS (Peng et al., 2014). *Schisandra chinensis* polysaccharide can regulate the levels of IFN-γ, TNF-α, IL-1β, IL-17, IL-13, IL-6, and other factors in colonic tissues, improve the diversity and composition of the intestinal microbiota as well as increase the levels of acetic acid, propionic acid, butyric acid and total SCFAs levels in UC mice (Su et al., 2020). *Atractylodes macrocephala* can change the composition of the intestinal microbiota, modulate the ability of the intestinal microbiota to produce SCFAs, and regulate the ability of the host and intestinal microbes to digest food nutrients, amino acids, and bile acid metabolism. (Feng et al., 2020). *Lonicera japonica* polysaccharide, the main active ingredient of *L. japonica*, can enhance the secretion of Secretory immunoglobulin A (sIgA) by promoting the secretion of the natural killer cell (NK) and cytotoxic T lymphocytes cytotoxicity, attenuate the apoptosis of splenic lymphocytes, restore the diversity of intestinal flora (Zhou et al., 2021).

### 4.2 Saponins

Saponins are particular glycosides widely found in the plant world and are named after their aqueous solutions that produce a large amount of soap-like foam that does not disappear after shaking. The chemical structure of saponins is complex and can be divided into steroidal saponins and triterpene saponins, depending on the chemical structure of the saponin elements produced after hydrolysis. Saponins have multiple physiological and biochemical activities and are widely found in plants (Dong et al., 2019).

#### 4.2.1 Baicalin

Baicalin is an active ingredient extracted from the dried roots of *Scutellaria baicalensis* Georgi (Wang et al., 2022). Baicalin significantly increased the activity of CAT, SOD, and GSH in colonic tissue of UC by blocking NF-κB and PI3K/Akt signaling pathways, reducing the release of IL-6, IL-1β, and TNF-α, increasing the level of IL-10, inhibiting TNBS-induced increase in ROS and MDA levels, control inflammatory responses and reduce oxidative stress damage (Shen et al., 2019; Zhu et al., 2020a). Baicalin effectively inhibits the expression of macrophage migration inhibitory factor (MIF), regulates macrophage function, promotes the proliferation of CD4^+^CD29^+^ cells, and regulates Th17/Treg balance. Furthermore, upregulating the expression level of Interferon regulatory factor 4 (IRF4) induces the differentiation of macrophages into M2-type macrophages with anti-inflammatory effects and alleviates the symptoms of colitis (Dai et al., 2012; Zhu et al., 2016; Zhu et al., 2020b). Baicalin can downregulate the expression levels of caspase-3, caspase-9, Bax, and Factor Related Apoptosis Ligand (FasL), promote the expression of Bcl-2, and effectively inhibit apoptosis (Zhu et al., 2020a). In terms of improving intestinal mucosal barrier function, Baicalin maintains the integrity of the intestinal mechanical and chemical barriers by downregulating the level of microRNA-191a, increasing the expression of occludin, ZO-1, and MUC-2, and reducing TNF-α-induced migration of IECs (Wang L. et al., 2017). It has also been shown that Baicalin can improve TNBS-induced dysbiosis of the UC intestinal flora by reducing the ratio of Firmicutes to Bacteroidetes, increasing the level of butyric acid, and regulating the metabolism of SCFAs (Zhu et al., 2020b).

#### 4.2.2 Naringin

Naringin, a compound extracted from fruits, has biological activities such as anti-inflammatory, anti-apoptotic, and anti-oxidative stress (Bharti et al., 2014), a potentially effective drug for IBD treatment. Naringin can reduce the inflammatory response in colonic tissues by upregulating the levels of SOD and GSH, reducing the levels of MDA and MPO, thus improving the TNBS-induced pathological changes in colitis (Cao et al., 2018; Hambardikar and Mandlik, 2022). In Acetic acid (AA)-induced colitis mice, Naringin restores the oxidative balance in the colonic mucosa by reducing the expression of Lactate dehydrogenase (LDH) and Alkaline phosphatase (ALP) in serum, thereby reducing the DNA damage (Kumar et al., 2014). Furthermore, in regulating the intestinal flora of UC mice, Naringin reduced the abundance of pathogenic bacteria to improve DSS-induced intestinal flora dysbiosis (Cao et al., 2021). Exploration of its potential mechanisms showed that Naringin could activate DSS-induced PPARγ, inhibit NF-κB, MAPK pathway, and NLRP3 activation, as well as regulate the expression of ZO-1 (Cao et al., 2018). These results suggest that Naringin may be a potential natural drug to improve DSS-induced UC symptoms in mice.

#### 4.2.3 Paeoniflorin

Paeoniflorin is a compound derived from *Paeonia lactiflora* Pall (Xin et al., 2019). that inhibits TNBS-induced production of inflammatory factors in the colon of UC mice to attenuate the inflammatory response (Gu et al., 2017). Paeoniflorin inhibits DCs by inhibiting the JAK/STAT signaling pathway, decreasing the level of IL-12 and the percentage of MHC-II^+^CD86^+^ DCs maturation, as well as restoring DC-mediated Th17/Treg homeostasis by reducing IL-17 secretion which upregulates Foxp3, IL-10 expression and induces the differentiation of naive T cells into CD4^+^CD29^+^ Treg cells (Zheng et al., 2020). Paeoniflorin significantly reduces eosinophil-associated levels of chemokines CCL11, CCL24, and CCL26, reducing eosinophil migration in the intestine and thus improving UC symptoms (Li J. et al., 2021). In regulating the composition of intestinal flora, Paeoniflorin can block the cytosolic dipeptide (Muramyl dipeptide, MDP)/NOD2 pathway, which in turn inhibits the nuclear translocation of NF-κB p65, increases the number of cupped cells, restores the crypt structure of the colon, reduces the infiltration area of pathogenic bacteria in intestinal tissue (Luo X. et al., 2021). In addition, in LPS-induced Caco-2 cells, Paeoniflorin restored the expression of TJ proteins such as claudin-5 and occludin and reduced the permeability of the intestinal epithelium by activating the Nrf2/HO-1 signaling pathway (Wu et al., 2019).

#### 4.2.4 Ginsenosides Rg1

Ginsenosides have been extracted from the genus Ginseng’s roots, stems, leaves, and fruits (Kang et al., 2021). Ginsenosides Rg1 can block the recognition of TLR, inhibit PI3K/Akt pathway transactivation and STAT protein activation, and regulate Follicular helper T cells (Tfh)/Treg cell homeostasis (Jin et al., 2021). Rg1 can target and regulate Neurite outgrowth inhibitor-B (Nogo-B)/Ras homolog gene family member A (RhoA) signaling pathway, adjust the polarization ratio of M1 and M2 macrophages, promote upregulation of NLRP12 expression, and suppresses IL-1β and TNF-α through TLR4 signaling pathway, further restoring the balance of anti-inflammatory and pro-inflammatory factors (Zhu et al., 2017; Long et al., 2022). In addition, Rg1 treatment improved the diversity of colonic microbiota in mice with colitis and effectively alleviated the symptoms of experimental colitis (Long et al., 2022).

#### 4.2.5 *Panax notoginseng* Saponin

*Panax notoginseng* saponins (PNS), a saponin-like component extracted from the dried roots and rhizomes of *Panax notoginseng* (Burkill) F.H.Chen, can be used to treat various diseases such as diabetes and atherosclerosis (Xu et al., 2019). PNS treatment was able to inhibit PI3K/Akt activation, increase IL-10 expression, significantly reduce the percentage of CD11BF4/80-labeled macrophages in DSS-induced SD rat colon tissue, and induced macrophage polarization to anti-inflammatory CD206^+^ M2 macrophages, thereby suppressing the intestinal inflammatory response (Lu et al., 2020). PNS inhibits the level of NO release from RAW 264.7 inflammatory cells induced by the triacylated lipopeptide Pam3CSK4 and the expression of inflammatory factors by targeting the p38 MAPK and TLR/NF-κB signaling pathways, with the attenuation of oxidative stress damage in the intestine (Luo H. et al., 2021). PNS inhibited the apoptotic response of the intestinal epithelium by increasing the expression of Bcl-2 and Bcl-3 and significantly downregulating the expression levels of caspase-3 and Bax (Lu et al., 2020). In addition, in restoring intestinal mechanical barrier function, PNS can alleviate intestinal mucosal barrier damage by increasing the expression of ZO-1, claudin-1, and occludin in colonic mucosal tissue (Lu et al., 2020).

#### 4.2.6 Astragaloside IV

Astragaloside IV is a natural saponin-like component extracted from *Astragalus membranaceus* (Fisch.) Bunge, with pharmacological effects such as asthma relief, anti-oxidative stress, and modulation of immune function (Li L. et al., 2017). Astragaloside IV can reduce the production of inflammatory factors in LPS-stimulated normal human colonic histiocytes by downregulating NF-κB signaling (Wu and Chen, 2019). In bone marrow-derived macrophages (BMDM), Astragaloside IV was able to regulate STAT3 signaling by inhibiting STAT1 activation, increasing the expression of M2 macrophage markers CD206, Ym1, and TGF-β, inhibiting pro-inflammatory M1 macrophage markers iNOS, IL-6, and IL-1β activity, decreasing M1 and M2 ratios and regulating macrophage function in the spleen (Tian et al., 2021). Another study showed that Astragaloside IV increased ATP content, stimulated nuclear translocation of β-Catenin, accelerated epithelial cell proliferation, improved the destruction of actin filaments, inhibited degradation of claudin-5 and occludin, which can effectively reduce the extent of damage to the colonic mucosa (Jiang et al., 2017). These results suggest that Astragaloside IV may be a new potential therapeutic approach for inflammatory bowel disease.

### 4.3 Alkaloids

#### 4.3.1 Matrine

Matrine is a natural piperidine alkaloid, the main active component of several legumes, with various pharmacological activities including anticancer, antiviral, and anti-inflammatory (You et al., 2020). Matrine can inhibit the colon by activating the antioxidant response of the Nrf2 pathway, suppressing the JAK2/STAT3 pathway, downregulating the expression of inflammatory factors, and regulating the levels of apoptosis-related factors epithelial cell apoptosis (Fang et al., 2018; Yao et al., 2021; Chen A. et al., 2022). Matrine treatment increases the expression of TJs protein and increases the expression of mucin-producing cells and MUC-2, protecting the intestinal barrier from DSS damage, which may involve the activation of the PPAR-α signaling pathway (Li et al., 2019). Oxymatrine is an alkaloid extracted from the root of bitter ginseng (Zhou et al., 2014) and can be used to treat a variety of acute or chronic inflammatory conditions. In DSS-induced UC mice, Oxymatrine can promote Treg differentiation by blocking the Rho protein RhoA/Rho-associated kinase (ROCK) signaling pathway and downregulating the ratio of Th1 to Th17 (Wang Y. et al., 2019). Oxymatrine can increase the levels of GSH and SOD in the colon and serum, inhibit the activity of myeloid MPO, iNOS, and COX-2, as well as the production of ROS, and effectively reduce oxidative stress (Tang et al., 2020). In addition, OMT can have a significant regulatory effect on apoptosis by blocking the PI3K/Akt signaling pathway and preventing DSS-induced downregulation of Bcl-2 and Bad with upregulation of caspase-3 and caspase-9 expression in colonic tissues (Chen et al., 2017). Oxymatrine treatment has a significant regulatory effect on apoptosis by blocking the TLR-9/MyD88/NF-κB pathway associated with downstream TNF-α, IL-1β, and IL-6 protein expression levels, restoring TJ protein expression and attenuating TNBS-induced colitis symptoms (Li et al., 2022).

#### 4.3.2 Berberine

Berberine is an isoquinoline alkaloid extracted from *Datura floribunda* Paxton. In TNBS-induced colitis, Berberine increases the expression of sIgA in the colon by downregulating STAT1 and STAT3 phosphorylation, inhibiting the NF-κB signaling pathway, decreasing Th1/Th17, and reducing pro-inflammatory cytokines, which can regulate the immune response homeostasis (Li et al., 2015). Berberine activates the PKB/SOCS1 signaling pathway, inhibits the phosphorylation of p65, reduces the polarization of pro-inflammatory M1 macrophages, and regulates the ratio of M1 and M2 type macrophages (Liu Y. et al., 2018). Berberine improves the damaged intestinal tract by activating the AhR signaling pathway, which promotes the expression of TJ epithelium (Jing et al., 2021). In addition, Berberine can restore carbohydrate digestion and absorption, glycolysis, gluconeogenesis, and amino acid metabolism by increasing the abundance of beneficial bacteria and decreasing the proportion of harmful bacteria (Liao et al., 2020). Furthermore, Berberine can also play a role in treating colitis by activating the Nrf2 pathway and inducing the expression of P-glycoprotein (Jing et al., 2018).

#### 4.3.3 Piperine

Piperine is one of the active components of the piper plant and has various pharmacological effects such as anti-inflammatory, antidepressant, and anti-gastric ulcers (Guo et al., 2020). Piperine treatment attenuates the inflammatory response of human colon cancer cells SW480 and HT-29 by blocking the MAPK signaling pathway and thus inhibiting the expression of chemokine CXCL8 (Hou et al., 2015). Piperine can inhibit the overexpression of ILs, TNF-α, COX-2, and iNOS by blocking the IκB-α/NF-κB signaling pathway. It can downregulate the expression of caspase-1 to control the inflammatory response and disease progression, inhibit apoptosis, ameliorate TNBS-induced reduction of TJs, and alleviate symptoms of colitis and protect intestinal epithelial cells (Guo et al., 2020). It has also been shown that Piperine may be a potential agonist and inducer of PXR, which can induce CYP3A4 gene expression of CYP450 enzyme lineage at the mRNA and protein levels, thereby preventing or reducing colonic inflammation (Hu et al., 2015).

#### 4.3.4 Sinomenine

Sinomenine is an alkaloid monomer extracted from *Cymbopogon ambiguus* (Hack.) A. Camus, which has been found to have pharmacological effects such as anti-inflammatory and immunomodulatory. It was shown that Sinomenine significantly improved the inflammatory response of intestinal mucosa in colitis, inhibited the secretion of IL-1β and TNF-α, and improved the severity of colitis in mice by downregulating the level of miRNA-155 and other related inflammatory cytokines (Yu et al., 2013).

### 4.4 Organic Acids

Organic acids have various components and chemical structures and are widely distributed in traditional Chinese medicine. Organic acids have the properties of general carboxylic acids, and most of them have particular acidity to produce esters, chlorides, amides, and other derivatives. They are not only part of the nutritional elements of living organisms but also play an essential role in human metabolism and display biological activity.

#### 4.4.1 Chlorogenic Acid

Chlorogenic acid is a compound found in various plants, fruits, and vegetables, which is synthesized from quinic acid by esterification with caffeic acid that exhibits anti-inflammatory, antiviral, antioxidant, and anticancer properties (Zatorski et al., 2015). Chlorogenic acid can downregulate MPO expression levels by inhibiting TLR4-mediated PI3K/Akt and NF-κB pathways, reducing NEUT infiltration and expression of pro-inflammatory cytokines in DSS-induced colitis as well as LPS-stimulated RAW 264.7 cells (Zhang P. et al., 2019; Chen L. et al., 2022). Chlorogenic acid can increase the expression of SOD1 and CAT, decrease MDA content and ROS production, and reduce oxidative stress damage (Wan et al., 2021). In addition, Chlorogenic acid attenuates apoptosis by inhibiting the expression of HO-1, Bax, caspase-8, and caspase-9 (Vukelic et al., 2018). Chlorogenic acid improves intestinal flora by regulating amino acid and lipid metabolism in SD rats, thereby reducing serum levels of LPS and promoting SCFA production, increasing the level of butyric acid, and reducing mucosal damage in the colon (Xie et al., 2021).

#### 4.4.2 Gallic Acid

Gallic acid is widely found in various fruits and plants, has anti-inflammatory, antioxidant, and antiviral effects (Zhu et al., 2019). Gallic acid can effectively inhibit the expression of p-IκBα and p-NF-κB in TNBS-induced UC and significantly increase the levels of IL-4 and IL-10 while down-regulating IL-6, IL-12, IL-17, IL 23, TGF-β and TNF-α expression, alleviating the inflammatory response of UC (Pandurangan et al., 2015b). Gallic acid also acts as a potent antioxidant that significantly upregulates Nrf2 with its downstream targets Uridine diphosphate-glucuronosyltransferase and NADH Quinone Oxidoreductase 1 (NQO1) in DSS-induced mice. Gallic acid further reduced the MPO, iNOS, and COX-2 in colonic tissues and improved mucosal damage in the intestinal epithelium (Pandurangan et al., 2015a).

#### 4.4.3 Ursolic Acid

Ursolic acid is a compound extracted from plants such as rosemary and fruit peels (Seo et al., 2018). Ursolic acid inhibits the activation of NF-κB and MAPK signaling pathways in IECs and macrophages, reduces TNF-α, IL-1β, COX-2, and iNOS in TNBS-induced colitis in mice as well as in LPS-stimulated inflammatory cells expression levels and attenuate the inflammatory response (Chun et al., 2014; Jang et al., 2014; Liu et al., 2016). Ursolic acid can inhibit the activation of JAK/STAT and c-Jun amino-terminal kinase (JNK) signaling pathways, prevent the excessive proliferation and differentiation of intestinal stem cells, and reduce ROS production in cells and alleviate damage to thylakoid cells (Wei et al., 2022). Ursolic acid can also reduce intestinal bacterial community abundance, regulate fatty acid metabolism, and affect immune cell infiltration and cytokine expression, which may be related to MAPK, IL-6/STAT3, AMPK/Forkhead Box Protein O (FOXO), and PI3K signaling pathways (Sheng et al., 2021).

#### 4.4.4 Rhein

Rhein is widely present in various herbal medicines (Wu et al., 2017). In UC mice, Rhein downregulates NF-κB and NLRP3 activity in macrophages and activates the Nrf2/HO-1/NQO1 pathway, inhibits NOX2 subunit expression, and translocation, downregulates protein expression levels of IL-6, IL-1β, TNF-α, iNOS, and COX-2, significantly reduces NO production. Rhein can increase the number of beneficial bacteria, decrease the proportion of pathogenic bacteria, alter the composition of the intestinal microbiota, and thus improve dysbiosis, which may be related to the inhibition of the PI3K/Akt/mTOR signaling pathway (Dong et al., 2022). Rhein can also indirectly regulate the level of uric acid metabolism in the intestine by increasing the fermentation products of *Lactobacillus* and restoring the barrier function of the intestine by decreasing intestinal permeability by increasing the expression of claudin-1, E-cadherin, and the secretion of mucus (Wu J. et al., 2020).

### 4.5 Flavonoids

Flavonoids are found in almost all green plants and have a wide range of biological activities.

#### 4.5.1 Luteolin

Luteolin is a flavonoid compound found in various plants with anti-inflammatory, anti-tumor, antibacterial, and anti-oxidative stress effects (Pandurangan and Esa, 2014). Luteolin can increase the expression of SOD and CAT in colon tissues, down-regulate MDA levels, enhance resistance to oxidative stress by activating the Nrf2 signaling pathway, and inhibit p-STAT1 and p-JAK1 expression, which in turn block the NF-κB pathway transduction. Luteolin can significantly reduce COX-2, iNOS, and IL-8 levels as well as slow NO overproduction, thereby ameliorating DSS-induced experimental colitis (Nunes et al., 2017). Luteolin can restore the function of the intestinal epithelial barrier by blocking the STAT3 signaling pathway and increasing the resistance values as well as the expression levels of ZO-1, claudin-1, and Polyclonal Antibody to Occludin (Li et al., 2016). Luteolin inhibits MAPK kinase (MEK) and ERK phosphorylation as well as decreases 5-hydroxytryptamine and tryptophan hydroxylase expression in a DSS-induced colitis model (Suga et al., 2021). Luteolin treatment also alters the diversity and composition of the gut microbiota in UC rats, mainly related to DNA repair, ribosome, purine, and pyrimidine metabolism (Li B. et al., 2021).

#### 4.5.2 Cardamonin

Cardamonin is a natural flavonoid that inhibits NO release, having anti-tumor and anti-platelet aggregation effects (James et al., 2021). In LPS-stimulated RAW 264.7 inflammatory cells, Cardamonin can exert anti-inflammatory effects by inhibiting the upregulation of TLR4 and MyD88 and reducing the expression of TNF-α and IL-6 (Ren et al., 2015). In human acute leukemia cells and mouse BMDMs, Cardamonin can reduce the production of associated pro-inflammatory factors by activating AhR, promoting the activation of the Nrf2/NQO1 signaling pathway, and inhibiting the activation of the NLRP3 (Wang K. et al., 2018). In addition, Cardamonin reduces caspase-3, MPO, iNOS, COX-2, and MDA, inhibits TNF-induced apoptosis, and reduces oxidative stress, improving AA-induced UC symptoms (Ali et al., 2017).

#### 4.5.3 Myricetin

Myricetin is a flavonoid extracted from prunes with anti-cell proliferation, antioxidant, anti-inflammatory, and anti-cancer effects (Zhang et al., 2018). In DSS-induced acute colitis in mice, Myricetin can regulate the immune response by promoting the stability of the internal environment of immune cells, increasing the ratio of CD4^+^CD29^+^ Treg, and restoring the Th17/Treg balance in mice (Qu et al., 2020). Myricetin can reduce the content of MPO and MDA by decreasing the production of NO, increasing the SOD and GSH expression, and exerting antioxidant effects in DSS-induced colitis (Zhao et al., 2013a). In terms of improving intestinal mucosal barrier function, Myricetin can increase the expression of claudin-1 and occludin, which restore the integrity of intestinal epithelial tight junctions (Qu et al., 2020). In addition, Myricetin can improve the intestinal microenvironment by improving the intestinal flora, and increasing the metabolism of ascorbic acid, aldehyde, and lipids (Miao et al., 2021).

### 4.6 Terpenoids

#### 4.6.1 *Tripterygium wilfordii* Hook F

*Tripterygium wilfordii* Hook F, whose main component is the diterpenoid rebaudioside (Zhang et al., 2016), has anti-inflammatory and immunosuppressive effects. *Tripterygium wilfordii* Hook F can regulate Th17/Treg imbalance in IBD patients (Ogino et al., 2013), which can also reduce microscopic inflammation modulate and inflammatory cytokines by enhancing the *in-situ* levels of Foxp3^+^+ Tregs (Li et al., 2014). It was found that *Tripterygium wilfordii* Hook F exerts anti-inflammatory effects by inhibiting NOXs-ROS-NLRP3 signaling pathway antioxidant and anti-lipid oxidation, thereby suppressing the expression of pro-inflammatory factors (Fangxiao et al., 2019). In addition, *Tripterygium wilfordii* Hook F has corticosteroid-like functions (Chen et al., 2005).

#### 4.6.2 Andrographolide

Andrographolide, a diterpene lactone, is the main active component of *Andrographis paniculata* (Burm.f.) Nees (Zhang L. et al., 2019). It has been shown that Andrographolide can activate the AMPK pathway, block the NF-κB and p38 MAPK signaling pathways, reduce NO production, and decrease the expression of iNOS and COX-2 (Kim et al., 2019). Andrographolide can improve the symptoms of DSS-induced acute colitis by regulating the STAT3 signaling pathway and decreasing the levels of factors such as IL-23, IL-17, and IFN-γ in serum and colon tissue, leading to a decrease in the percentage of Th1 and Th17 cells in CD4^+^ cells, which in turn promotes the anti-inflammatory response of Th2 (Zhu et al., 2018). In addition, Andrographolide can improve oxazolone (OXA)-induced UC symptoms by blocking the IL-4R/STAT6 signaling pathway, reducing the specific binding of IL-4/IL-13 to IL-4R, and inhibiting MPO activity and TNF-α secretion (Zhang L. et al., 2019).

## 5 Conclusion

The role of TCM and natural products is gaining more and more attention, just as the great potential in treating COVID-19 patients has been revealed. The general public concept of TCM may be a mixture of several plants, while TCM should be a mixture of natural products in nature. Both TCM and Western medicine originate from plants. With the development of chemical science, western medicine has stepped out of using a single compound as the active ingredient, while TCM is still in the mixture stage. The role of TCM is mainly reflected in the regulation of human gene activity, while western medicine directly acts on protein targets. Natural products from plants are regulators of human gene activity, and the role of TCM at the gene level is mainly to regulate the activity of human genes. Western medicine has a clear target, most of which are enzymes or receptor proteins, so Western medicine works at the protein level. TCM and Western medicine often have good complementarity, and clinical combination therapy can achieve a better therapeutic effect.

The biggest problem in TCM development is that the efficacy cannot be quantified, the methodology and standards have not been unified, and the markers of TCM quality control have not been clear. Based on practical experience, only by genuinely studying the mechanism of action of TCM from the perspective of biology and establishing a quantitative system of TCM efficacy can TCM gradually enter the palace of quantitative science, and TCM science will usher in a new situation.

The development of IBD is the result of “multiple strikes.” Therefore, immune deficiency, intestinal flora disorders, and increased intestinal permeability often characterize patients. Biological agents are often used in Western countries for IBD patients, costing thousands of dollars per year to treat. Many patients cannot afford such expensive treatment. In recent years, the Chinese medicine industry has developed rapidly. Driven by modern technology, more and more researchers have begun to contact TCM and have a strong interest in it. The TCM and natural products, as mentioned above, can regulate immune cells, immune-related pathways, intestinal flora, improve intestinal barrier function, regulate oxidation, autophagy, and angiogenesis. TCM and natural products are multi-pathways and multi-targets, which can produce more effective and comprehensive therapeutic effects on IBD under the holistic and evidence-based view of Chinese medicine. The study of TCM and natural products can further clarify the components and mechanisms of drug action. In future clinical treatment, we can start from the above directions to discover drugs that can effectively treat IBD. However, the related research is still at the introductory stage, and more in-depth analysis is needed. In addition, the composition of TCM is complex, and research on the active components of TCM and their mechanisms of action should be strengthened to rationalize the use of drugs and enrich the theoretical basis of clinical use of TCM. Although the diversity of TCM limits its clinical application, it is believed that with the development of clinical pharmaceutical technology, new high-efficiency, and low-toxicity formulations may bring new development space for TCM in the treatment of IBD. Therefore, TCM for IBD treatment still needs further improvement and in-depth research. In future research, emphasis should be placed on systematic and comprehensive treatment, and modern advanced scientific and technological means should be used to promote TCM-related research so that TCM can better benefit patients.

## Glossary

IBD: Inflammatory Bowel Disease
CD: Crohn’s Disease
UC: Ulcerative Colitis
TCM: Traditional Chinese Medicine
Th: helper T cells
Tregs: Regulatory T cells
Tfh: T follicular helper: Follicular helper T cells
IL: Interleukin
TGF: Transforming Growth Factor
DCs: Dendritic Cells
IFN: interferon
TNF-α: Tumor Necrosis Factor-α
iNOS: inducible nitric oxide synthase
NF-κB: Nuclear Factor Kappa B
IκB: inhibitor of NF-κB
PXR: pregnane X receptor
TLRs: TOLL-like Receptors
MyD88: myeloid differentiation factor 88
DSS: Dextran Sulfate Sodium
JAK: Janus Kinase
STAT: Signal Transducer and Activator of Transcription
NLRP: NOD-like Receptor Protein
ASC: Apoptosis-associated Speck-like protein Containing a CARD
caspase-1: cysteine aspartate protease 1
PPAR: peroxisome proliferator-activated receptor
SCFAs: Short-Chain Fatty Acids
TJ: Tight Junction
ZO: Zonula Occludens proteins
MLCK: Myosin Light Chain Kinase
IECs: Intestinal Epithelial Cells
ROS: Reactive Oxygen Species
SOD: Superoxide Dismutase
CAT: Catalase
GSH: Glutathione
MPO: Myeloperoxidase
MDA: Malondialdehyde
Nrf2: Nuclear factor E2-related factor 2
ATG: autophagy-related genes
VEGF-A: vascular endothelial growth factor-A
HIF-1α: hypoxia-inducible factor-1α
MCP: monocyte chemotactic protein
PDGF-BB: platelet-derived growth factor-BB
MMP: matrix metalloproteinase
TNBS: 2,4,6-Trinitrobenzene Sulfonic acid
APS: *Astragalus membranaceus* polysaccharides
DOPS: Dendrobium officinale polysaccharides
sIgA: Secretory immunoglobulin A
NK: natural killer
MIF: migration inhibitory factor
FasL: Factor Related Apoptosis Ligand
LDH: Lactate dehydrogenase
ALP: Alkaline phosphatase
MDP: Muramyl dipeptide
Tfh: T follicular helper: Follicular helper T cells
Nogo-B: Neurite outgrowth inhibitor-B
RhoA: Ras homolog gene family member A
PNS: *Panax notoginseng* saponins
BMDM: bone marrow-derived macrophages
ROCK: Rho-associated kinase
NQO1: NADH Quinone Oxidoreductase 1
JNK: c-Jun amino-terminal kinase
FOXO: Forkhead Box Protein O
MEK: MAPK kinase
OXA: oxazolone
